# Response: Collective Moral Agents and Their Collective-Level Virtues

**DOI:** 10.1093/phe/phac008

**Published:** 2022-04-09

**Authors:** Kathryn MacKay

**Affiliations:** Sydney Health Ethics, The University of Sydney, Australia

## Abstract

In this short piece, I attempt to respond to some of the challenges raised by Jessica Nihlén Fahlquist and Karen Meagher in their commentaries on my paper, ‘Public Health Virtue Ethics’. While these authors have made many insightful and challenging remarks, I mostly focus on two questions here: first, about the nature of collectives as moral agents, in response to Nihlén Fahlquist, and second, about the concept of a collective-level virtue, in response to Meagher.

In the two commentaries presented in this symposium, Jessica Nihlén Fahlquist and Karen Meagher present stimulating and challenging responses to my paper on public health virtue ethics. It is very rewarding to be in dialogue on this topic. I am extremely grateful to both authors for taking the time to read and respond to my paper as carefully as they have.

My paper represents a tentative initial account of structures of virtue in the context of public health practice, and Nihlén Fahlquist and Meagher have each identified weaknesses that need attention. The concerns raised by Nihlén Fahlquist and Meagher show, which questions are perhaps most pressing in developing this account further.

The table included in Meagher’s commentary, which employs David Pear’s taxonomy of virtue-related goals, provides a useful way to compare the accounts of virtue in public health that I, Michael Rozier and Nihlén Fahlquist present. While my account is focussed on collectives as such, Nihlén Fahlquist’s work in this area has focussed on public health practitioners as individuals, and Rozier has focussed on community members as individuals. It is the collective focus of my account that Nihlén Fahlquist most closely critiques, and I begin with her concerns.

Nihlén Fahlquist takes me to task for not addressing questions around conceptualizing public health as a group moral agent in this paper. Early on, I set this issue aside because giving an account of group moral agency for public health requires a full paper of its own. But, because the issue is pressing and Nihlén Fahlquist is right to say so, I will make a brief response.

What do we mean when we assign moral agency to a collective or group? We do assign moral agency to collectives and groups, with regularity, but this does not make the answer to this question easy to furnish (Collins, 2019). Nihlén Fahlquist asks, is public health primarily the sum of individual actions and activities reflecting individual agency, or is it primarily a collective actor with some kind of group agency (5)?

The answer to this partly depends on how we have defined public health. The metaphysical complexity of ‘public health’ has been written about previously, and it is clear that there are many meaningful ways to interpret this phrase (Dawson and Verweij, 2007). The ambiguity of the term ‘public health’ calls for a distinction between (at least) acts that can be characterized as contributing to the public’s health, acts done in the name of the health of the public and acts done in the offices of an agency with some special responsibilities or roles regarding the health of the public. Though many things can fall under the umbrella of ‘public health’, I was limiting my discussion to public health as a branch of government in welfarist societies. Examples of such governmental branches include the Public Health Agency of Canada, or Public Health England, which holds a range of official roles and powers in a democratic state.

Defined as a branch of the government, the discussion of public health in my paper excludes some of the examples that Nihlén Fahlquist includes at the end of her commentary, such as the physician talking with a patient about weight loss. Ordinary clinical encounters, characterized by a physician or treatment team aiming to benefit an individual patient, do not count as public health (Wilson, 2021: 10). It is possible that the physician’s conversation could be in line with an overarching Public Health Agency endeavour to decrease obesity rates, but as a general practitioner, the doctor may not be specifically affiliated with a public health agency nor acting in a public health capacity. So, physician–patient encounters can sometimes be characterized as contributing to public health without being public health actions. Certainly, the physician–patient relationship is focussed on the health of the individual patient, and not on the whole of the public.

Even defined this way, though, the question remains: is a Public Health Agency, as a branch of a government, a sum of the individuals who make it up and carry out its initiatives or is it primarily a collective? If a group has moral agency, what does this mean?

I have posited that public health so defined can be conceptualized as a collective with moral agency, and again in this response, I cannot fully explore this idea. However, Nihlén Fahlquist is mainly worried that if we look at public health as primarily a collective with moral agency, then we will imagine that practices and social structures exist without individuals (6). This is a problem because public health initiatives can be harmful to individuals, as a variety of historically recent and distant examples demonstrate. I would emphasize that Nihlén Fahlquist and I are both concerned about the power that public health holds, and my account does not endorse the action public health undertakes just by virtue of conceiving public health as a collective agent.

My response to this worry, then, is that the analysis of public health as a group moral agent is not an endorsement of everything it does; in fact, it helps us to locate and diagnose systemic harms that can arise within unexamined public health practices. Viewing public health agencies as moral agents gives us an additional tool for analysing public health practices and initiatives, and explaining some of the ways in which public health gets things wrong. My discussion of stigma and the ability of public health agencies to undermine inter-group relations is an example of how considering virtue at this level can add to the critical power of public health ethics. While Daniel Daly describes, and I reiterate, that practices can *appear* as objective reality, that is an explanation of the kind of power that practices come to hold over the individuals in society, and not an endorsement of those practices. This should give us pause and asks public health to reflect upon the use of its power to shape the social context.

That said, thinking of public health as a collective moral agent is only a model, a conceptual tool. As Midgley (1992: 147) says, ‘a model is only a model …’; we ‘need to keep correcting one model philosophically against another’ for a concept of social life to begin to take shape. Public health ethics will surely need to keep the individual agent in view, alongside public health agencies conceptualized as collective agents, to form the most robust picture of public health activity in a society.

Turning to the second commentary, Karen Meagher rightly highlights a slippage in my paper between the referent of virtue being public health structures (e.g. compassionate policies), and the referent of virtue being a social attribute (e.g. policies which cultivate compassionate societies). These are connected and it seems to me that virtuous policies will contribute to creating virtuous societies. However, I acknowledge that I should have kept the distinction between these clearer, not least of all because *how* a policy can be virtuous and *how* it could then foster social virtue are each complex questions to explore.

What I’d like to address in this response is something that Meagher presses me on and which I am also keen to better understand. This is the nature of collective virtues, specific to public health. A preliminary question Meagher asks is why it might be a *distinctive* goal of public health to structure societal virtues, such as compassion. She remarks, ‘it is unclear to me why public health institutions should seek to create inter-group compassion any more than any other public or civic institution’ (4). Then there is a further set of questions around how we should conceptualize virtues as features of collective practices or social structures.

My initial response to Meagher’s first question is that public health may not be solely responsible for structuring virtue in a society. It is likely that, as Meagher suggests, public health shares this responsibility with other civic organizations or agencies. However, and importantly, public health as I’ve defined it in the paper has unique abilities to support or undermine virtues insofar as public health has some powers that are not shared by other civic organizations or governmental branches: e.g. the police powers widely exercised during the coronavirus disease 2019 (CoVid-19) pandemic.

Examples of the ways in which exercising these powers undermine inter-group relations abound. In one example from Australia, in July 2021, New South Wales Health (the state department responsible for public health) placed strict restrictions on movement for people living within three metro-Sydney local government areas (LGAs) in response to there being 111 local cases of CoVid-19 (Razik, 2021). Residents of Canterbury-Bankstown, Liverpool and Fairfield were banned from leaving their LGAs unless they were health or emergency services workers. The mayors of these areas expressed frustration and feelings of exclusion and hurt on behalf of their communities. The restrictions placed on these LGAs, while others continued to enjoy relative freedom, increased distrust of public health (for seeming to be ill-founded or arbitrary) and undermined feelings of cooperation and solidarity. Outside the LGAs, the measures undermined compassion for residents of these three areas by blaming them for an outbreak, and singling them out as problematic and in need of a different set of restrictions from everyone else.

In exercising some powers, then, it seems clear that public health agencies can damage or undermine social virtues like compassion, friendship or justice. As such, we should hold public health accountable for this. In turn, public health should avoid having this effect as much as possible, and actively strive to foster virtues through its policies and practices. The bottom line here is that public health may not be the only governmental agency with a role in fostering virtue in a society, but it does have unique powers that can be used to undermine or uphold structures of virtue.

There is a related and difficult question Meagher asks, which has to do with establishing a collective account of virtues. She asks, for example, ‘when we say that we desire collective trust in public health, do we mean that public health institutions can be trustworthy in the same way as individuals? Or do we mean the kind of trust we need is the same as the figurative use of the expression, as in “I trust that the sun will rise tomorrow” (i.e., I believe I can count on things to be the same)? Or do we mean that organizations are trustworthy in an entirely third way, distinct to institutions (5)?’.

These are important questions to which I have not yet found answers. I have a hypothesis that there is something different about collective-level virtues when compared to individual-level virtues, but developing an account of this is part of my ongoing project. As an example, I think that the virtue of friendship fully conceived at the collective level (what I think of as ‘civic friendship’) might look different from the individual virtue of friendship in some significant ways, but also share some characteristics.

To wit, civic friendship might be vaguer and more diffuse than friendship (the individual virtue), involving a general feeling of goodwill and a tendency towards loose cooperation with others in your society, even if they are unknown to you on a personal level. There may be some shared traits between civic friendship and friendship, including a tendency to show partiality towards some people over others; a sense of goodwill towards particular others; and some level of desire for the other to do well. Different traits between civic friendship and friendship might include interpersonal distance rather than intimacy; loose connections between my doing well and your doing well; and loose connections between my character and yours.

It also seems like a collective virtue of justice would be different from an individual virtue of justice (which Aristotle was a bit vague on himself). While many virtues seem most at home in the individual character, justice seems more naturally to fit at the collective level than the individual, and I am eager to work out what a fully conceived collective virtue of justice would look like. These are not my fully formed views on the question, but suggestions about how collective-level virtues might have some shared qualities as well as differences when compared to the individual-level virtues with which we are familiar.

To tie this to Meagher’s first question, too, while public health may share responsibility for creating the structures of justice in a society with other organizations or branches of government (meaning it might not be a *distinctive* goal of public health against other branches), public health has unique powers to contribute to or undermine social justice. Additionally, if it is not a distinctive goal of public health over other organizations or branches, justice (often conceptualized as equity among practitioners) is at least a very important or even central goal for public health.

To conclude, I wish to express again my gratitude for the time and effort that Jessica Nihlén Fahlquist and Karen Meagher put into crafting their helpful, insightful and challenging commentaries. They have given me a lot to think about, and I hope to continue this dialogue with them, and others, on the topic of public health virtue ethics. As is clear from this symposium, there is much work to do in this area.
